# High incidence of superficial and deep medial collateral ligament injuries in ‘isolated’ anterior cruciate ligament ruptures: a long overlooked injury

**DOI:** 10.1007/s00167-021-06514-x

**Published:** 2021-03-04

**Authors:** Lukas Willinger, Ganesh Balendra, Vishal Pai, Justin Lee, Adam Mitchell, Mary Jones, Andy Williams

**Affiliations:** 1 Fortius Clinic, 17 Fitzhardinge St, London, W1H 6EQ UK; 2 Department of Trauma and Orthopaedics, Chelsea and Westminster Hospital, Chelsea and Westminster NHS Foundation Trust, London, UK; 3 Department of Orthopaedic Sports Medicine, Technical University of Munich, Ismaninger Straße 22, 81675 Munich, Germany

**Keywords:** Anteromedial rotatory instability, Medial collateral ligament, Magnetic resonance imaging, Deep MCL injury, Anterior cruciate ligament

## Abstract

**Purpose:**

In anterior cruciate ligament (ACL) injuries, concomitant damage to peripheral soft tissues is associated with increased rotatory instability of the knee. The purpose of this study was to investigate the incidence and patterns of medial collateral ligament complex injuries in patients with clinically ‘isolated’ ACL ruptures.

**Methods:**

Patients who underwent ACL reconstruction for complete ‘presumed isolated’ ACL rupture between 2015 and 2019 were retrospectively included in this study. Patient’s characteristics and intraoperative findings were retrieved from clinical and surgical documentation. Preoperative MRIs were evaluated and the grade and location of injuries to the superficial MCL (sMCL), dMCL and the posterior oblique ligament (POL) recorded. All patients were clinically assessed under anaesthesia with standard ligament laxity tests.

**Results:**

Hundred patients with a mean age of 22.3 ± 4.9 years were included. The incidence of concomitant MCL complex injuries was 67%. sMCL injuries occurred in 62%, dMCL in 31% and POL in 11% with various injury patterns. A dMCL injury was significantly associated with MRI grade II sMCL injuries, medial meniscus ‘ramp’ lesions seen at surgery and bone oedema at the medial femoral condyle (MFC) adjacent to the dMCL attachment site (*p* < 0.01). Logistic regression analysis identified younger age (OR 1.2, *p* < 0.05), simultaneous sMCL injury (OR 6.75, *p* < 0.01) and the presence of bone oedema at the MFC adjacent to the dMCL attachment site (OR 5.54, *p* < 0.01) as predictive factors for a dMCL injury.

**Conclusion:**

The incidence of combined ACL and medial ligament complex injuries is high. Lesions of the dMCL were associated with ramp lesions, MFC bone oedema close to the dMCL attachment, and sMCL injury. Missed AMRI is a risk factor for ACL graft failure from overload and, hence, oedema in the MCL (especially dMCL) demands careful assessment for AMRI, even in the knee lacking excess valgus laxity. This study provides information about specific MCL injury patterns including the dMCL in ACL ruptures and will allow surgeons to initiate individualised treatment.

**Level of evidence:**

III.

## Introduction

Rotatory knee instability is a major cause of morbidity for patients. Besides cruciate ligament tears, it is related to disruption of the peripheral medial and lateral soft tissue envelope, and meniscal lesions [9, 13, 18, 24, 31]. Anterolateral rotational instability (ALRI) has been extensively studied over the last decade due to interest in the anterolateral soft tissues. Conversely, the medial collateral ligament complex has received surprisingly little attention, especially with regards to anteromedial rotatory instability (AMRI) in combination with a cruciate ligament injury. This is surprising for a number of reasons. First, combined injuries to the anterior cruciate ligament (ACL) and the medial collateral ligament complex (MCL) comprise the most common two-ligament injury of the knee [40]. Second, it is our experience that surgeons often think MCL injuries are often considered benign and that it is thought the vast majority of patients do well with non-operative management, but this is not always true. There is evidence of increased failure of ACL reconstruction with unaddressed, even mild, MCL laxity [1, 36]. There is a lack of appreciation in the role of the MCL complex in resisting axial rotation as well as valgus stress, and therefore, significant injuries to the MCL complex do not only result in valgus, but also rotational laxity excess. Recent work has highlighted the importance of the MCL complex in the context of ACL injury with dMCL failure leading to increased external rotation laxity and AMRI [3, 5, 38, 39]. In addition, problems with healing of isolated deep MCL (dMCL) injuries have previously been established [26]. MCL laxity, therefore, is a spectrum from pure valgus to pure axial rotation excess and usually a combination.

During an injury causing apparently clinically ‘isolated’ ACL rupture, subluxation of the knee joint occurs, stretching the surrounding soft tissue envelope and causing damage to the structures concerned. Magnetic resonance imaging (MRI) studies have previously evaluated the impact on the anterolateral soft tissues in detail [4, 6, 8, 11, 16], but studies have not examined the medial soft tissue injuries to the same depth, nor specifically to the level of the MCL’s component parts, i.e. superficial MCL (sMCL), dMCL, and posteromedial capsule/posterior oblique ligament (POL).

The purpose of this study was to investigate the MCL complex injury patterns, their incidence, and the risk factors for them in clinically ‘isolated’ ACL injures. It was hypothesised that a true single ligament ACL injury (with or without meniscal pathology) is a rare phenomenon, and that associated MCL complex injuries occur in the majority of cases. A higher grade of anterior drawer/Lachman test, and bone oedema adjacent to the dMCL femoral attachment were believed to be risk factors for dMCL injuries. This study is the first that looks at specific MCL injury patterns including the dMCL in ACL ruptures and will allow surgeons to initiate individualised treatment.

## Materials and methods

Ethical approval to undertake the study was given by the institution involved (Fortius Clinic, London, UK) in line with the UK Health Research Authority guidance. This retrospective cohort study is comprised of a consecutive series of patients who underwent ACL reconstruction for MRI and arthroscopically confirmed clinically complete ‘isolated’ ACL rupture between Sep 2015 and Apr 2019. For the purposes of this study, the term ‘isolated ACL injury’ refers to a knee in which the only clinically significant ligament laxity was in regards to the ACL, but includes cases with concomitant meniscal pathology. Only patients who had MRI scans, performed to a minimal standard, within 3 weeks of ACL injury were included in the study, to allow for accurate evaluation of damage to the peripheral structures. Patients with previous ipsilateral knee injury, history of reconstructive surgery, or any abnormal ligament clinical examination findings, apart from ACL laxity, were excluded. This was to enable study of the full ligamentous injury in so-called ‘isolated ACL injured knees’. Patients with concomitant meniscus lesions were not excluded.

### Data collection

A retrospective review of medical records of all patients who had an isolated ACL reconstruction between Sep 2015 and Apr 2019 was performed. Patients eligible for inclusion in the study were identified and information regarding age, gender, mechanism of injury, time from injury to MRI and surgery and intraoperative findings were obtained. ACL graft failure was assessed at final follow-up after a minimum of 12 months.

### Clinical assessment

All patients were routinely examined under anaesthesia (EUA) at the beginning of surgery by the senior author who is a specialist sports knee surgeon with over 20 year of experience. EUA included a thorough knee ligament assessment (anterior and posterior drawer, Lachman, pivot-shift test, dial test, valgus and varus stress tests). The findings of Lachman, anterior drawer, and valgus/varus stress tests were categorised according to International Knee Documentation Committee (IKDC) form (grade I: 3–5 mm, grade II: 5–10 mm, grade III: > 10 mm) as laxity differences compared to the healthy contralateral knee [10]. Dial test was also graded according to IKDC form (grade 1: 6–10°, grade 2: 11–19°, grade 3: > 20°) in 30° and 90° knee flexion. The pivot shift test was graded as 0 (normal), 1 (glide), 2 (clunk), or 3 (subluxation).

### Radiological assessment

Preoperative MRI examinations were performed within the first 3 weeks after injury. The images were acquired from multiple centres. Minimum imaging criteria for inclusion included (1) field strength of 1.5 T or above, (2) 3-plane (sagittal, axial and coronal) imaging using water sensitive fat-suppressed sequences (STIR, fat-suppressed proton density or T2-weighted) and (3) slice thickness of 3 mm or less. As the images were acquired from multiple units, other sequences were often included such as T1-weighted imaging, but for the purpose of the study, and to maintain consistency, only the fluid sensitive sequences were used for image analysis.

Two radiologists (J.L. and A.M.) specialised in musculoskeletal imaging with 20 and 25 years of experience, independently analysed all MRI images and recorded the status of the sMCL, dMCL and POL injuries for each patient. In addition, the presence or absence, and the location of bone oedema was recorded. Bone oedema was defined as increased signal intensity on the fat-suppressed water sensitive images. Specific attention was paid to focal bone marrow oedema within the peripheral medial femoral condyle centred on the non-articular cortex at the deep MCL attachment.

**Fig. 1:**
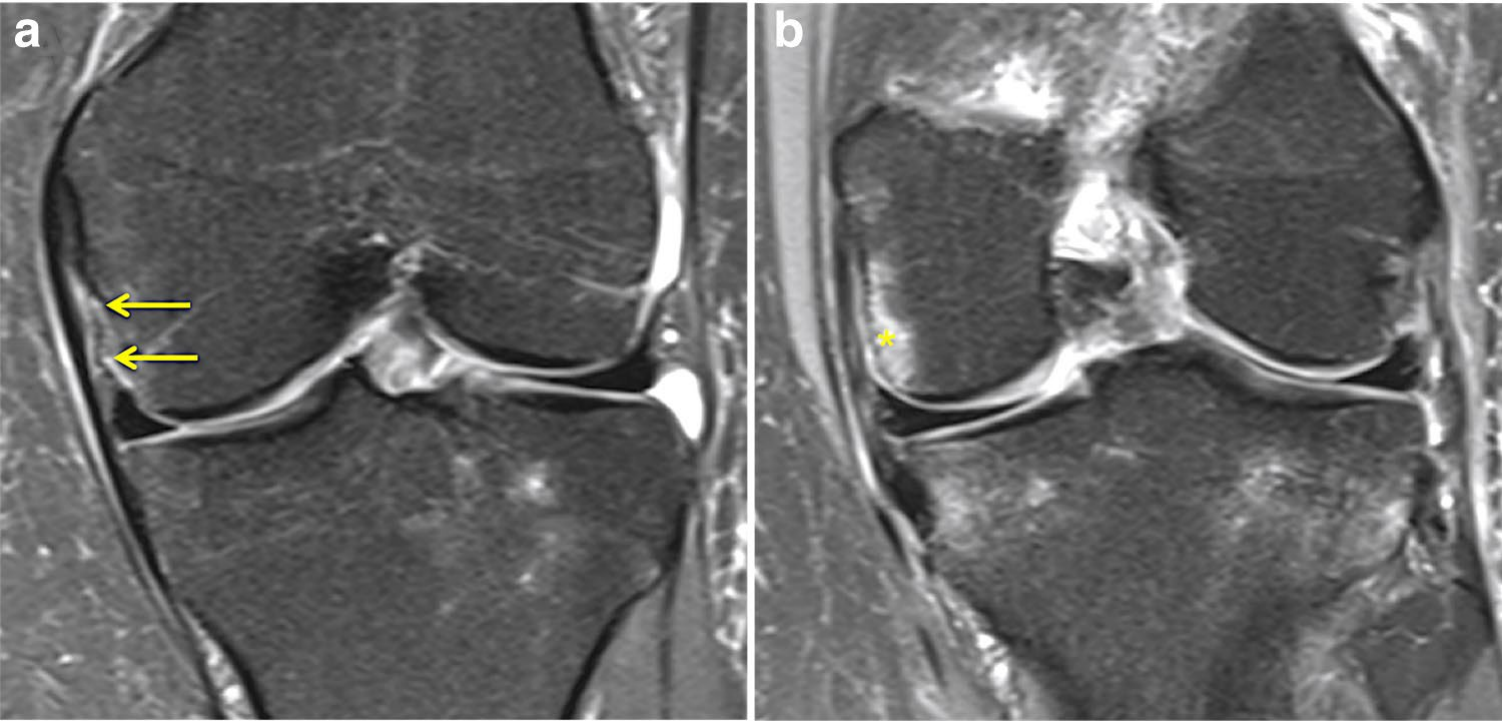
Sequential coronal fat-suppressed proton density-weighted magnetic resonance imaging shows **a** a meniscofemoral dMCL injury accompanying an ACL rupture with presence of joint fluid between the dMCL and the superficial MCL (yellow) and **b** a typical MRI bone oedema at the medial femoral condyle adjacent to the dMCL attachment site (*)

The sMCL injuries were classified according to a MRI based description from Rasenberg et al. [27]: grade I: peri-ligamentous swelling with minor tearing of sMCL fibres without complete disruption; grade II: complete disruption of superficial layer; and grade III: as grade II plus additional fluid extravasating from the joint into peri-ligamentous tissue. Any sMCL injury location was reported as femoral attachment, intra-ligamentous, or tibial attachment. The dMCL was classified as torn when continuity of their fibres was clearly disrupted or joint fluid was evident between the deep and superficial MCL fibres (Fig. 1). The injury location was documented as either meniscofemoral or meniscotibial. The POL was assessed and any oedema documented. The technique used in this study for measurement of medial and lateral tibial slope has been previously published and validated by the other authors [14, 21, 25]. The slope was defined as the difference between the tibial joint orientation and a perpendicular line to the proximal anatomical tibial axis. A positive value indicates a steeper posterior tibial slope. Medial–lateral slope asymmetry was calculated by subtracting the medial tibial slope from the lateral tibial slope.

### Statistical analysis

Data were analysed using SPSS statistics software version 23.0 (IBM, New York, NY, USA). Normal distribution was confirmed by the Shapiro–Wilk test and continuous variables were expressed as mean ± standard deviation. Chi-square test or Fisher’s exact test was used to analyse for any association between MCL injuries and demographic variables, EUA and MRI findings. Binomial logistic regression analysis was performed to evaluate the risk factors for the presence of dMCL injuries. The included predictive factors were age, sex, injury mechanism, medial and lateral tibial slope, tibial slope asymmetry, anterior drawer, Lachman, and pivot shift tests, and presence of sMCL injury and bone oedema at the medial femoral condyle (MFC) close to the dMCL attachment site. Cohen’s kappa value has been calculated for inter-rater agreement and inter-rater correlation coefficient (ICC) was calculated for inter-rater reliability between the two readers. A post hoc power analysis to identify differences in number of dMCL injuries in intact versus damaged sMCL patients resulted in an actual power of 87.2% with a critical *p* value of 0.05 (G*Power 3.1). Statistical significance was set at a *p* value of < 0.05.

## Results

Hundred patients (80 male and 20 female) with a mean age of 22.3 ± 4.9 years and a minimum follow-up of 12 months (mean 35.6 ± 11.4) were included. There were 81 non-contact and 19 contact injuries in 53 right and 47 left knees. The time between injury and MRI was 2.5 ± 3.2 days (range 0–21) and 16.6 ± 13.1 days (range 3–100) between injury and surgery. All included patients were professional athletes to ensure MRIs were obtained shortly after injury: 60 soccer players, 26 rugby players, 4 ballet dancers and 10 players from other sports (netball, hockey, lacrosse, cricket, gymnastics, handball, and swimming). This group was chosen as early MRI scanning is routine.

### Clinical assessment

Preoperative EUA showed a positive anterior drawer test in all patients: 83% grade I, 17% grade II. Lachman tests were graded I in 2%, II in 35% and III in 63% of the patients. Pivot shift tests were positive in all patients: 29% grade I, 54% grade II and 13% grade III. Owing to the inclusion criteria, posterior drawer, valgus and varus stress tests were negative in all patients, as was the dial test.

### Incidence of medial collateral ligament complex injuries

MRI examination revealed that 67% of patients had concomitant injuries to their medial ligament complex that was not evident on clinical examination, and only 33% returned normal signal. There were 29% isolated sMCL, 4% isolated dMCL injuries, 23% combined sMCL/dMCL, 7% combined sMCL/POL, 3% combined sMCL/dMCL/POL, and 1% combined dMCL/POL injuries.

The majority of sMCL injuries were classified by MRI as grade I (91.9%) lesions, and the remaining were grade II (8.1%). sMCL tears were typically found to be intra-ligamentous (72.6%), but 22.6% occurred at the femoral origin and 4.8% at the tibial insertion. Of the 31% of patients with dMCL injuries, 93.4% occurred at the meniscofemoral part and only 6.4% at meniscotibial part. The POL was only abnormal in 11% of patients and was never injured in isolation.

Cohen’s kappa showed excellent agreement between the two readers for the assessment of sMCL lesions (0.926, *p* < 0.001) and dMCL lesions (0.953, *p* < 0.001). The ICC values for reliability of MRI measurements were 0.942 (95% CI 0.915–0.961) for sMCL injuries and 0.954 (95% CI 0.932–0.969) for dMCL injuries also indicating excellent agreement.

### Factors associated with MCL injuries

The association between the patients’ EUA results and concomitant injuries and the presence of any medial ligament complex injury (sMCL, dMCL and/or POL) or injuries involving the dMCL (isolated or with other parts of the MCL complex) are summarised in Tables 1, 2.

**Table 1:** Patient’s EUA results in relation to the presence of medial ligament complex injury or a deep medial collateral ligament (dMCL) injury

		Medial ligament complex		*p* value	dMCL		*p* value
		Intact	Injured		Intact	Injured	
Anterior drawer test	Grade I	23 (27.7%)	60 (72.3%)	***0.022***	54 (65.1%)	29 (34.9%)	n.s.
	Grade II	10 (58.8%)	7 (41.2%)		15 (88.2%)	2 (11.8%)	
Lachman test	Grade I	1 (50%)	1 (50%)	n.s.	1 (50%)	1 (50%)	n.s.
	Grade II	11 (31.4%)	24 (68.6%)		25 (71.4%)	10 (28.6%)	
	Grade III	21 (33.3%)	42 (66.7%)		43 (68.3%)	20 (31.7%)	
Pivot shift test	Grade 1	12 (41.4%)	17 (58.6%)	n.s.	23 (79.3%)	6 (20.7%)	n.s.
	Grade 2	18 (33.3%)	36 (66.7%)		38 (70.4%)	16 (29.6%)	
	Grade 3	3 (23.1%)	10 (76.9%)		6 (46.2%)	7 (53.8%)	

Data are given as numbers (percentage)

**Table 2:** Concomitant injuries in relation to the presence of medial ligament complex injury or a deep medial collateral ligament (dMCL) injury

		Medial ligament complex		*p* value	dMCL		*p* value
		Intact	Injured		Intact	Injured	
sMCL injury	Intact	n/a	n/a		33 (86.8%)	5 (13.2%)	***< 0.001***
	Grade I	n/a	n/a		36 (63.2%)	21 (36.8%)	
	Grade II	n/a	n/a		0 (0%)	5 (100%)	
POL	Intact	n/a	n/a		62 (69.7%)	27 (30.3%)	n.s.
	Oedema	n/a	n/a		7 (63.6%)	4 (36.4%)	
Medial meniscus	Intact	24 (36.4%)	42 (63.6%)	n.s.	47 (71.2%)	19 (28.8%)	n.s.
	Tear	9 (26.5%)	25 (73.5%)		22 (64.7%)	12 (35.3%)	
Lateral meniscus	Intact	18 (40.0%)	27 (60.0%)	n.s.	31 (68.9%)	14 (31.1%)	n.s.
	Tear	15 (27.3%)	40 (72.7%)		38 (69.1%)	17 (30.9%)	
Intraoperative ramp lesions	Absent	33 (39.3%)	51 (60.7%)	***0.001***	63 (75.0%)	21 (25.0%)	*** < 0.01***
	Present	0	16 (100%)		6 (37.5%)	10 (62.5%)	
MFC bone oedema adjacent to dMCL attachment	Absent	28 (38.9%)	44 (61.1%)	n.s.	58 (80.6%)	14 (19.4%)	*** < 0.001***
	Present	5 (17.9%)	23 (82.1%)		11 (39.3%)	17 (60.7%)	

Data are given as numbers (percentage)

**Fig. 2:**
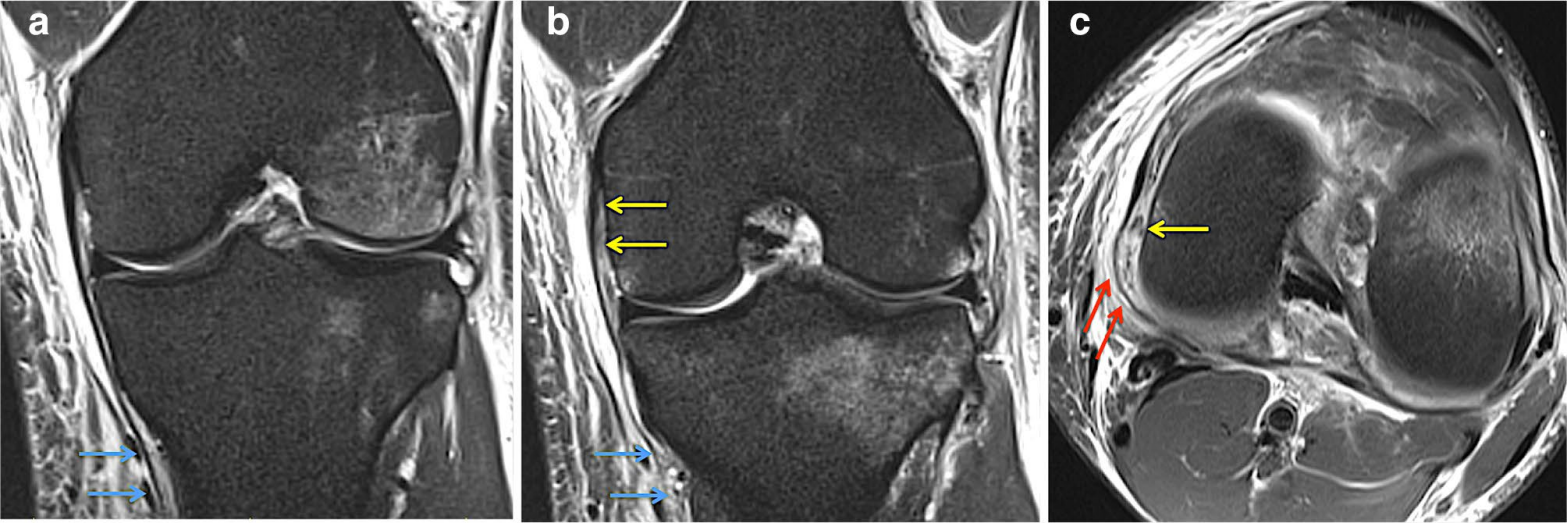
Sequential coronal (**a**, **b**) and axial (**c**) fat-suppressed proton density-weighted magnetic imaging shows a tibial superficial MCL lesion MRI grade II (blue), a deep MCL lesion at the meniscofemoral portion (yellow) and a disruption of the posteromedial capsule and POL (red) with surrounding soft tissue oedema

**Fig. 3:**
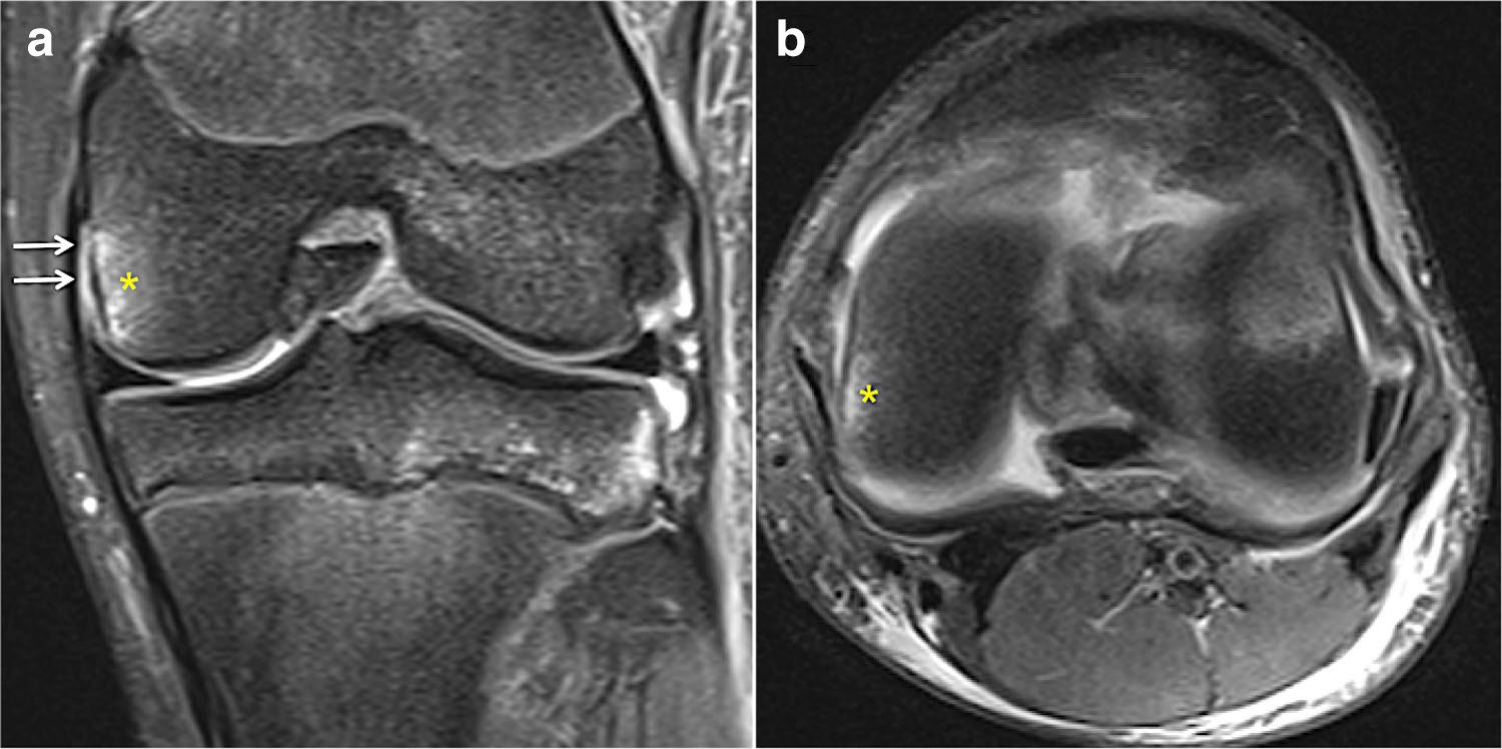
Coronal (**a**) and axial (**b**) fat-suppressed proton density-weighted magnetic resonance imaging indicates a dMCL injury (white) with bone oedema (*) at the medial femoral condyle adjacent to the dMCL attachment

A medial ligament complex injury was associated with the intraoperative finding of medial meniscus ramp lesion (*p* = 0.001) and it was also negatively related to an anterior drawer test grade II outcome (*p* < 0.01). A dMCL injury was significantly associated with sMCL tears on MRI and present in 100% of MRI sMCL grade II injuries (the worst grading in this series) (OR 4.767, 95% CI 1.639–13.859, *p* < 0.001, Fig. 2). It was also associated with an intraoperative finding of a medial meniscus ramp lesion (OR 5.000, 95% CI 1.621–15.419, *p* < 0.01) and especially with the appearance of bone oedema at the MFC adjacent to the dMCL attachment site (OR 6.403, 95% CI 2.459–16.671, *p* < 0.001, Fig. 3). There was no significant correlation between medial ligament complex or dMCL injuries and age, injury mechanism, medial or lateral tibial slope and tibial slope asymmetry.

### Risk factors for dMCL injury

Logistic regression analysis with backward elimination identified the risk factors associated with the presence of dMCL injuries (Table 3). Of eleven initially included predictor variables only three increased the risk of dMCL lesion significantly: age, presence of sMCL injury, and bone oedema at the MFC adjacent to the dMCL attachment site. Age decreased the risk of sustaining a dMCL injury significantly by 1.2 times with every year of age. The risk of exhibiting a dMCL injury was significantly raised by a simultaneous sMCL (OR 6.75) and by the presence of bone oedema at MFC adjacent to dMCL attachment (OR 5.54). The prediction model was statistical significant (*χ*^2^ (4) = 32.688, *p* < 0.001) and explained 40.9% of the variance in the presence of dMCL injuries (Nagelkerke, *R*^2^). This model correctly classified 80.2% of patients. The sensitivity was 55.2%, specificity was 91.0%, positive predictive value was 72.7% and negative predictive value was 82.4%. The area under the curve for the prediction model was 0.808 (95% CI 0.720, 0.896), which is an excellent level of discrimination. The characteristic bone oedema at the MFC adjacent to the dMCL attachment seems very closely linked to dMCL lesions.

**Table 3:** Logistic regression analysis shows the association between the presence of a deep medial collateral ligament injury and clinical and radiological factors

Factor	Odds ratio	95% CI	B ± SE	*p* value
Age	0.846	0.735 –0.974	− 0.167 ± 0.072	*** 0.020***
Anterior drawer test	0.197	0.029 –1.354	− 1.623 ± 0.983	n.s.
sMCL injury	6.750	1.793–25.412	1.909 ± 0.676	*** 0.005***
MFC bone oedema	5.539	1.848 –16.598	1.712 ± 0.560	*** 0.002***
Constant value			1.064 ± 1.556	n.s.

Odds Ratio describes the risk of exhibiting a dMCL injury. Nagelkerke *R*^2^ = 0.409. Area under the curve is 0.808 (95% CI 0.720, 0.896)

*CI* confidence interval, *sMCL* superficial medial collateral ligament, *MFC* medial femoral condyle

### Postoperative outcomes

Two patients (2%) sustained an ACL graft re-rupture after a minimal follow-up of 12 months. The ACL graft failure was neither correlated to sMCL lesion (1/2 case) nor dMCL lesion (0/2 cases).

## Discussion

The main finding of this study is the high incidence of MCL complex injury (67%) with a high number of sMCL (62%) and dMCL injuries (31%) in a so-called ‘isolated’ ACL rupture. In addition, the presence of bone oedema at the MFC adjacent to the dMCL attachment site, MRI grade II sMCL injury and ramp lesions are highly correlated with dMCL injury. These findings were despite normal clinical examination findings for MCL laxity.

As early as 1968, in a pre-MRI era, Slocum and Larson identified the importance of a combined ACL/MCL injury and related it to the existence of abnormal anterior displacement of the medial tibial plateau (which equates to external tibial rotation with an axis in the lateral compartment of the knee) causing AMRI [33]. The injury mechanism for the combined ACL/MCL injury was described as a combined valgus and external rotation load to the knee with successive injury to the ‘medial capsular ligament’ (dMCL), the sMCL and the ACL [15, 33]. Recent biomechanical studies support this theory and have emphasised the importance of the dMCL in resisting external tibial rotation [3] and shown a significant increase in anteromedial laxity after the sMCL or dMCL has been cut [5, 28, 38]. Despite its importance, there has been little study of the dMCL. A recent anatomical study describes the dMCL as a fan-shaped structure, passing from a small femoral attachment, just posterior and distal to the sMCL femoral attachment on the medial epicondyle, to a wider tibial attachment [2]. It is overall orientated obliquely, especially the anterior portion, passing anteriorly as well as distally [2, 39]. This obliquity means the dMCL tightens when the tibial is externally rotated, making it ideal to resist anterior translation of the medial tibial—i.e. AMRI [3, 39], analogous to the concept of the anterolateral ligament [7] on the lateral side for ALRI. The dMCL is the primary restraint to external rotation, whilst the sMCL is the primary restraint to valgus [3, 19, 28]. MCL deficiency is known to cause a large increase in ACL tension when the knee is loaded with either valgus or external rotation torques [23, 29]. This is clinically reflected in a higher ACL re-rupture rate in ACL/MCL combined injuries when the MCL was left unaddressed compared to intact or surgically treated MCLs [1, 36].

The present study, as with similar MRI studies of the lateral soft tissues in ACL rupture, confirm that injuries to the soft tissue envelope are common. The incidence of 67% MCL complex injury in the present study is higher in comparison to previous reports, where the incidence of concomitant MCL injuries with ACL ruptures has ranged from 22 to 44% [12, 17, 20, 30, 34]. This can probably be explained by the short time from injury to MRI assessment at an average of 2.5 days in the present study. Our findings support a previous report, which showed that the dMCL can be subject to isolated injury leaving the other parts of the MCL uninjured. These can be problematic especially amongst soccer players and can require surgery [26]. In the case series by Narvani et al. [26], during clinical examination external rotation precipitated the pain the players presented with, and dMCL injury can reveal increased external rotation even with an intact ACL. In addition to the dMCL, external rotation is resisted by the posterior horn of the medial meniscus acting rather like a ‘wheel-chock’ against the medial femoral condyle [35]. Therefore, the impact of a dMCL injury can be offset, at least for a while with an intact medial meniscus. However, over time, such repetitive loading often leads to failure of the posterior part of the medial meniscus which then results in exacerbation of AMRI [22, 33]. In addition, in the acute setting, this may explain the association between ramp lesions and dMCL injuries found in our study.

In the present study, the documented MCL injuries did not result in increased valgus laxity of the medial ligament complex and were, therefore, of little clinical concern. Nevertheless, their frequency, in the absence of clinical MCL laxity, implies that more major medial injuries, which might be problematic in terms of causing further instability symptoms, are likely to be more common than realised. Occult, clinically important MCL injury is made even more likely by the fact that whilst surgeons routinely assess laxity on valgus stress (due to sMCL lesions), they do not assess for AMRI. Indeed in their 1968 publication Slocum and Larson stated: ‘Although long recognised as a clinical entity, rotatory instability has too often been overlooked by inexperienced surgeons because standard testing methods stress valgus rocking and anteroposterior joint laxity rather than rotatory instability’ [33]. The lack of routine clinical testing for AMRI means it is likely that cases of AMRI are under-appreciated and these ‘missed’ cases could lead to ACL graft stress overload and account for some cases of failure of ACL grafts [23, 29]. The challenge is to identify cases with excess MCL laxity and especially AMRI, and select them for concomitant MCL surgery when ACL reconstruction is undertaken. Whilst MRI is reliable at showing soft tissue injury, it is unreliable in assessing the degree of disruption, therefore, appropriate clinical testing will be of more value [32].

Whilst the dial test can be positive with AMRI, it is undertaken with a focus on posterolateral rotatory instability (PLRI) and so it is likely some subtle cases of AMRI are overlooked. In the publication by Slocum and Larson [33], clinical AMRI was defined clinically as a pathologically increased forward displacement of the medial tibia plateau when the tibia is pulled anteriorly (an anterior drawer) whilst 15° externally rotated at 90° knee flexion (having excluded ALRI). Surprisingly, the results of the present study showed an inverse relationship between grade of anterior drawer test and MRI confirmed MCL injury. This seems odd when it might be thought that increased anterior drawer laxity would correlate with bigger injury overall including to the medial soft tissue envelope; however, and in contrast to the publication by Slocum and Larson, anterior drawer testing was done with the foot in a neutral position rather than in 15° external tibial rotation. The standard anterior drawer is less relevant than a Lachman test to examine the ACL [37] as secondary restraints such as the posterior horn of the medial meniscus contribute more resistance to anterior tibial translation at 90° compared to 30°. In view of the discussion above regarding the importance for also testing for external rotation excess (AMRI) perhaps the Slocum test described above should be routinely undertaken.

Whilst the need for anteromedial stabilisation surgery is likely to be less frequently needed than additional anterolateral surgery, it may, however, be advantageous in a significant number of ACL reconstruction cases. The authors of the present study recognise the scenario that some knees, when examined after ACL graft fixation, despite no excess ACL laxity, a negative pivot shift, and no excess valgus laxity, seem to have some abnormal anteromedial tibial laxity. In such cases pre-operative awareness of MCL (specifically dMCL), MRI abnormality is beneficial, as it will alert the clinician to take a much closer clinical evaluation to rule out occult AMRI, and hopefully less of these cases that compromise ACL reconstruction will be missed, and appropriate surgical procedures, which need future development, can be employed.

This study exhibits limitations. All patients were professional athletes and do not represent a typical patient cohort. Their knees may be subject to different levels of force compared to recreational athletes or layperson. However, this group was specifically chosen as it ensured early MRI examination (average 2.5 days after injury), thereby permitting prompt and comprehensive assessment of acutely injured knees and their medial soft tissue injury patterns. MRIs were acquired in various institutions with different protocols. Therefore, minimum imaging requirements have been applied for inclusion to allow for reliable analysis, which was reflected by high inter-rater agreement. This is the first MRI study that differentiates the separate component parts of the medial ligament complex (sMCL, dMCL, POL). This is important as each has a different role in resisting excess motion [3, 28]. The dMCL is especially pertinent to AMRI as it resists anterior translation of the medial tibia/external rotation. Oedema in soft tissue alone might not be necessarily indicative of a disruption of ligament fibres, but may indicate injury to adjoining tissue with the resultant spread of oedema. This is especially relevant to POL injuries, which were mainly identified by soft tissue oedema rather than clear ligament disruption, and was never found to be injured in isolation. The relevance of POL injury in combination with ACL is thus questionable.

It must be emphasised that, despite the high number of concomitant MCL injuries in ‘isolated’ ACL injuries, these findings should not lead to unnecessary and poorly indicated surgery. The results from the present study underline the importance of clinical examination to detect AMRI, the need to develop more focussed examination techniques, and also to raise the awareness of medial soft tissue injury in the context of ACL rupture, which may be a cause, in some cases, of ACL graft compromise. These findings provide the first description of distinct MCL injury patterns and shall help surgeons to make a precise diagnosis, individualised treatment and prevent persistent rotatory instability and re-injuries. Further clinical studies are needed to facilitate surgeon’s decision-making in patients with AMRI.

## Conclusion

Regarding ligamentous injury of the knee, a truly ‘isolated’ ACL rupture is uncommon. 67% have an MCL complex injury, with 62% of these involving the sMCL and 31% the dMCL. The presence of bone oedema at the MFC adjacent to the dMCL attachment site, MRI grade II sMCL injury and age were identified as significant risk factors for having a dMCL injury. Ramp lesions were correlated with the presence of a dMCL injury. Since missed AMRI is a risk factor for ACL graft failure, oedema in the MCL (especially dMCL) demands careful scrutiny for occult AMRI, even in the knee lacking excess valgus.
